# Recognizing a duplicated inferior vena cava during radical nephrectomy for papillary renal cell carcinoma: A case report

**DOI:** 10.1016/j.eucr.2026.103651

**Published:** 2026-10-23

**Authors:** Muhammad Wajid Siddique, Muhammad Waqas, Muhammad Hanzla Shahzad, Muhammad Hassaan Javaid, Nazila Dalir

**Affiliations:** a Ameer-ud-din Medical College, Lahore, Pakistan; b Shaukat Khanum Memorial Cancer Hospital and Research Centre, Lahore, Pakistan; c Shifa College of Medicine, Islamabad, Pakistan; d School of Medicine, St. George's University, St. George's, Grenada

**Keywords:** Duplicated inferior vena cava, Papillary renal cell carcinoma, Renal cell carcinoma, Radical nephrectomy, Venous anomaly, Surgical planning

## Abstract

Duplication of the inferior vena cava (DIVC) is a rare congenital venous anomaly with important implications during retroperitoneal surgery. A 40-year-old man presented with 3-month hematuria and mild left flank pain. CT revealed a 7-cm left renal mass, pulmonary and osseous lesions, and two separate IVC channels near the aortic bifurcation. PET/CT confirmed DIVC and suspected metastases. Open radical nephrectomy with lymph-node dissection was performed without vascular injury. Histopathology showed papillary renal cell carcinoma, ISUP grade 3, confined to the renal capsule. This case highlights the importance of recognizing DIVC before renal surgery.

## Highlights

• Duplicated inferior vena cava (DIVC) is uncommon and may increase the risk of venous injury during renal and retroperitoneal surgery.
• Careful preoperative review of contrast-enhanced CT can identify venous variants and facilitate safer operative planning.
• In this patient, DIVC was confirmed intraoperatively during radical nephrectomy for papillary renal cell carcinoma.

## 1 Introduction

Duplicated inferior vena cava (DIVC) is caused by persistence of fetal venous systems and is a rare congenital vascular malformation. The meta-analysis estimated its total prevalence at about 0.7%, although there is considerable variability among the population and methods of detection.^1^ Asymptomatic DIVC is detected by accident in cross-sectional imaging studies. However, DIVC remains unrecognized before retroperitoneal surgery and can cause iatrogenic venous injuries during hilar, para-aortic, lymph-node, or ureteric dissections 2, 3, 4, 5.

Papillary renal cell carcinoma (PRCC) is one of the main variants of non-clear cell RCC and may be detected with advanced stages due to relatively insignificant symptoms. Cabozantinib demonstrates antitumor effect in advanced PRCC and significantly improves progression-free survival in comparison with sunitinib in the PAPMET trial.^6^ Here we describe a case of a patient with papillary RCC and metastatic disease in which preoperative diagnosis of DIVC was done and DIVC was confirmed during surgery.

## 2 Case presentation

A 40-year-old male with no prior comorbidities had three months of hematuria and mild left flank pain. His physical exam was unremarkable for an abdominal mass. Table 1 below shows the chronology of the case.

**Table 1:** Chronology of events in the case.

Time/date	Clinical event
∼3 months before presentation	Hematuria and mild left flank pain; physical examination unremarkable.
Preoperative work-up	Outside contrast-enhanced CT: heterogeneous left lower-pole renal mass, bilateral pulmonary nodules, and two venous structures adjacent to the infrarenal aorta suggesting DIVC.
6 Jan 2026	PET/CT: hypermetabolic left renal tumor, bilateral lung nodules, and osseous lesions involving T8 and sacrum.
19 Jan 2026	Open left radical/cytoreductive nephrectomy with retroperitoneal lymph-node dissection through a left subcostal incision; DIVC identified intraoperatively; no reported vascular injury.
Postoperative pathology	Papillary RCC, ISUP grade 3, 70 mm, pT1b; intact cortical capsule; negative renal sinus/perinephric fat, ureteric and vascular margins; two reactive lymph nodes.
Postoperative period	MRI thoracic spine: altered marrow signals at D8, D12 and L1; no extraosseous soft-tissue component or canal narrowing.
∼2 months postoperatively (March 2026)	Clinically well; cabozantinib started. Nivolumab was being arranged, with plan to continue nivolumab/cabozantinib until progression or unacceptable toxicity.

Contrast enhanced CT scan showed a heterogeneous lesion of 7 cm originating from the lower pole of the left kidney. There were multiple bilateral pulmonary nodules, which were suspected to be metastatic. There were two venous channels near the infra-renal aorta, and one on the right side is a normal IVC, whereas the other one was suspected to be a duplicated IVC (Fig. 1).

**Fig. 1:**
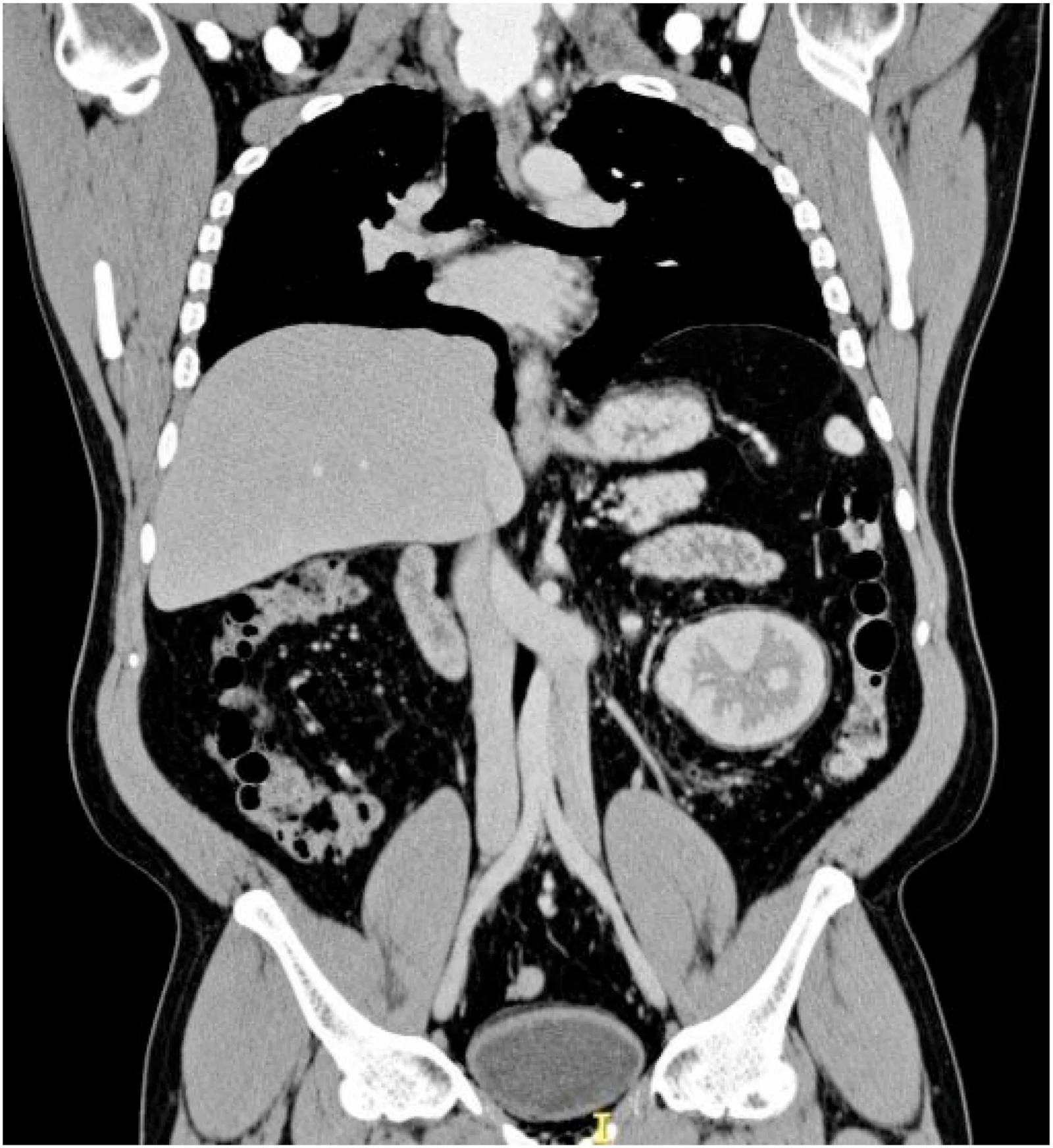
Preoperative Coronal Contrast Enhanced CT image showing the heterogeneous left renal mass. The CT image also shows two venous channels next to the infra-renal aorta, suggesting duplicated IVC.

The PET/CT done on 6th January 2026 showed a hypermetabolic heterogeneously enhancing tumor in the left renal cell carcinoma, hypermetabolic pulmonary nodules bilaterally and osseous lesions involving the T8 vertebra and sacrum, suggesting metastatic disease. Postoperatively, the MRI of the thoracic spine showed altered marrow signal in D8, D12 and L1 levels without extraosseous soft tissue involvement or spinal canal narrowing.

Laboratory investigations revealed persistently increased erythrocytosis with microcytosis. On 21st January 2026, Hb was 18.3 g/dL, Hct 55%, RBC count 7.19 × 10^6^/μL, MCV 76.5 fL. Hemoglobin remained elevated in repeated tests, with a value of 18.9 g/dL on 4th March 2026. Given the context of renal cell carcinoma, the patient's erythrocytosis was believed to be paraneoplastic, but the erythropoietin levels and workup for primary erythrocytosis was not done (Table 2).

**Table 2:** Selected hematologic laboratory results.

Parameter	Reference range	21 Jan 2026	23 Jan 2026	22 Feb 2026	4 Mar 2026
WBC (×10^3^/μL)	4.52–10.93	11.04	9.01	10.61	9.99
RBC (×10^6^/μL)	4.54–5.59	7.19	6.93	7.42	7.64
Hemoglobin (g/dL)	13.2–16.7	18.3	17.5	18.4	18.9
Hematocrit (%)	40.1–49.0	55.0	53.6	55.8	57.5
MCV (fL)	77.8–96.2	76.5	77.3	75.2	75.3
RDW-CV (%)	11.9–15.2	18.0	17.4	18.7	18.8
Platelets (×10^3^/μL)	150–450	278	219	204	251

Following a multidisciplinary consultation, the patient proceeded with left open radical nephrectomy with retroperitoneal lymph-node dissection on 19 January 2026 via left subcostal incision. There was a large lower pole renal mass seen. There was one renal artery and renal vein. The duplicated IVC was noted in the retroperitoneal operative field consistent with the preoperative CT-suspected venous anomaly (Fig. 2).

**Fig. 2:**
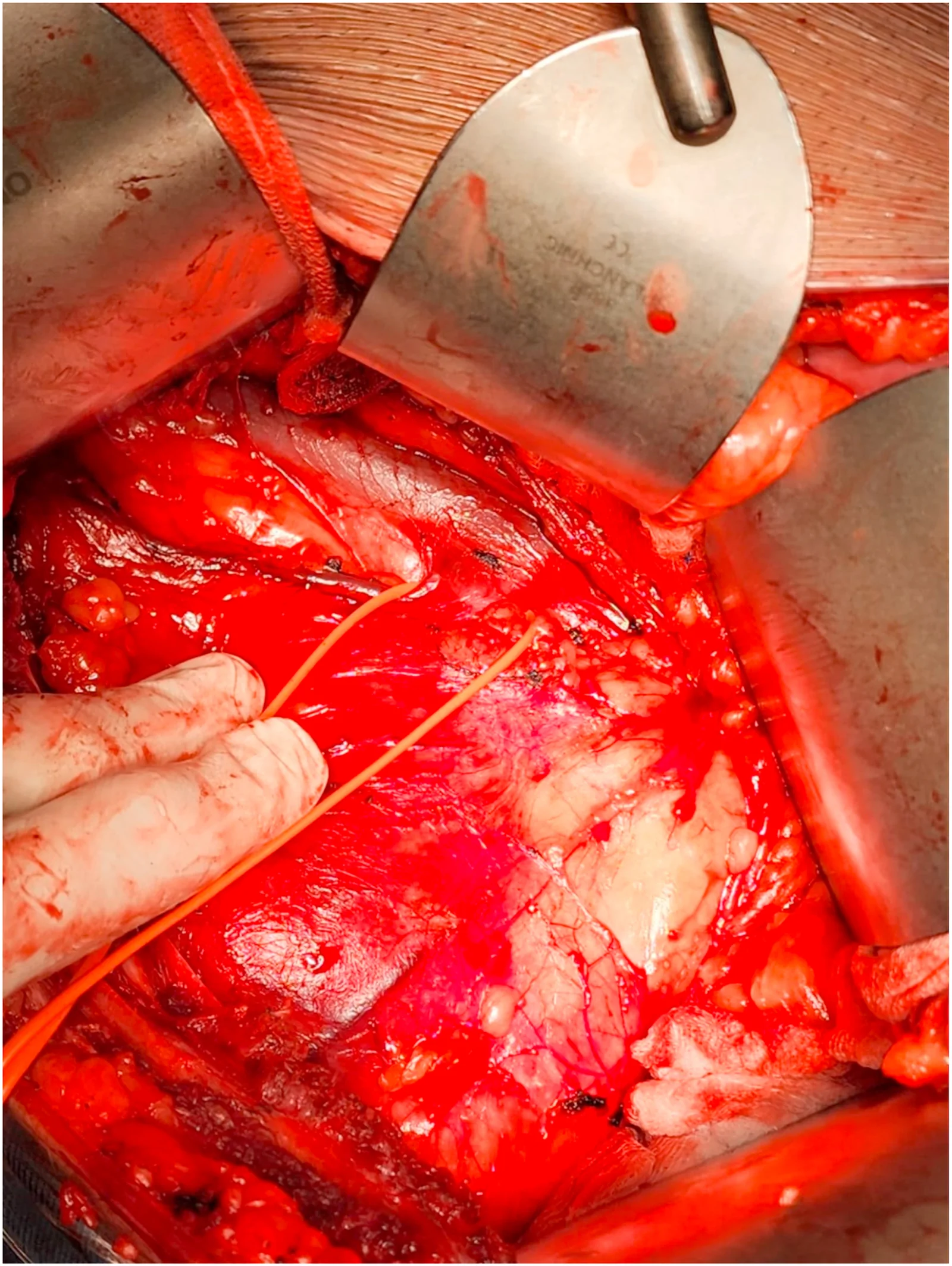
Intraoperative picture during open radical nephrectomy showing the retroperitoneal operative field where the duplicated IVC is noted.

There was mobilization of the colon and dissection of the renal hilum. The renal artery was then ligated and divided, followed by ligation and division of the renal vein. Mobilization of the kidney was done circumferentially, keeping the adrenal gland in situ, with division of the ureter. An enlarged retroperitoneal lymph node was resected by sharp dissection. Hemostasis was achieved by application of Surgicel. There was no reported vascular injury or intraoperative complication.

Histopathologic examination revealed renal cell carcinoma, papillary, ISUP grade 3 measuring 70 mm in size. Tumor is limited by an intact cortical capsule without invasion of the renal sinus and perinephric fat. Ureteric and vascular margins are negative. Two sampled retroperitoneal lymph nodes are reactive and free of malignancy.

Thus, the primary lesion was histopathologically staged as pT1b. But taking into account preoperative pulmonary and osseous findings, the clinical picture suggests metastatic RCC in spite of the pT1b pathology of the nephrectomy specimen.

Postoperative recovery was uneventful. The patient was doing well clinically about two months postoperatively. Cabozantinib was started for advanced papillary RCC, while nivolumab was being prepared for combination therapy. The next oncology treatment strategy was systemic therapy until progression or toxicity.

## 3 Discussion

DIVC is a rare congenital venous anomaly associated with persistence of embryological venous channels. According to a meta-analysis, the prevalence is about 0.7%.^1^ Embryologically, persistence of the left supracardinal venous system can give rise to the left-sided infrarenal venous channel which usually joins the left renal vein and then communicates with the right-sided IVC.^2^, ^3^, ^5^

The main clinical significance of DIVC in this particular case is associated with the possibility of intraoperative complications. The anomalous venous channel can be confused with the lymphatic system, gonadal vein, collateral vein or some other structure located in the retroperitoneum and injury can lead to considerable hemorrhage which can complicate the renal hilar or para-aortic dissection.^4^, ^5^ Earlier studies stressed the significance of the venous anomalies (particularly of IVC and renal venous anomalies) in renal surgery, including nephrectomy of RCC.^7^, ^8^

In the current patient, the DIVC was detected in the preoperative contrast-enhanced CT and then confirmed intraoperatively. This case illustrates the practical principle of importance: when two venous structures are detected in the vicinity of the aorta, it is necessary to study their course, size, tributaries, relation to the renal veins, confluence. Multidimensional reconstruction of the image and visualization in three dimensions can be particularly useful in unclear cases.^4^, ^5^ Although the DIVC itself does not require treatment, identification of the anomalous venous system prior to surgery increases the preparedness of the surgeon.

Oncological considerations also require attention. This patient had the 7-cm, ISUP grade 3 papillary RCC with pulmonary and osseous lesions suspicious of metastases. It is necessary to distinguish between the pathological tumor stage and overall clinical stage of the disease: the specimen was pT1b and radiological signs indicated the clinical stage M1 disease. The metastatic lesions were not pathologically confirmed in the provided information and it is possible to regard the metastatic lesions only as clinically suspected metastases.

Persistent erythrocytosis is one more important point to consider. Renal cell carcinoma can cause paraneoplastic erythrocytosis due to the production of inappropriate erythropoietin by the RCC.^7^ However, taking into account the presence of microcytosis, it is impossible to rule out the other causes of this condition and it is impossible to determine the cause of the anemia (because the erythropoietin and molecular examination was not conducted).

Treatment of advanced PRCC with cabozantinib is evidenced. According to the PAPMET study, the median progression-free survival for patients treated with cabozantinib is 9.0 months, while for sunitinib-treated patients the median progression-free survival is 5.6 months.^6^, ^9^ Combination therapy of cabozantinib and nivolumab proved to be effective for papillary, unclassified, and translocation-associated RCC with the objective response rate of 47.5% and median progression-free survival of 12.5 months.^10^, ^11^ Thus, the patient's systemic management reflects contemporary treatment approaches for advanced non-clear-cell RCC.

## 4 Conclusion

DIVC is a rare but important form of venous abnormality that may have significant ramifications when performing renal and retroperitoneal surgeries. In this case of papillary RCC, the venous abnormality was identified before surgery through the CT scan, and the diagnosis was confirmed intraoperatively without any harm done to the veins. This particular case highlights the importance of systematic analysis of venous structures of the retroperitoneum as an integral part of preoperative preparation for renal surgery. Correct identification of the number of veins, their course, and convergence prevents inadvertent venous injuries and facilitates safer surgical dissection.

## CRediT authorship contribution statement

**Muhammad Wajid Siddique:** Conceptualization, Formal analysis, Project administration, Methodology, Supervision, Validation, Writing – original draft, Writing – review & editing, Data curation. **Muhammad Waqas:** Writing – original draft. **Muhammad Hanzla Shahzad:** Writing – original draft, Writing – review & editing. **Muhammad Hassaan Javaid:** Writing – original draft. **Nazila Dalir:** Writing – original draft.

## Informed consent

Written informed consent for publication of the case and accompanying images was obtained from the patient.

## Ethics approval

Ethical approval was not required for this single case report according to institutional policy.

## Patient perspective

The patient reported anxiety regarding his cancer diagnosis and surgery but was reassured following successful surgery and postoperative recovery.

## Data availability statement

All case-specific data relevant to this report are contained within the manuscript.

## Funding

No specific funding was received.

## Conflict of interest

The authors declare no conflicts of interest.
